# Nicotine Motivated Behavior in *C. elegans*

**DOI:** 10.3390/ijms25031634

**Published:** 2024-01-29

**Authors:** Chinnu Salim, Enkhzul Batsaikhan, Ann Ke Kan, Hao Chen, Changhoon Jee

**Affiliations:** Department of Pharmacology, Addiction Science and Toxicology, College of Medicine, University of Tennessee Health Science Center, Memphis, TN 38163, USA; chinnusalimbc@gmail.com (C.S.);

**Keywords:** nicotine, motivated behavior, *C. elegans*

## Abstract

To maximize the advantages offered by *Caenorhabditis elegans* as a high-throughput (HTP) model for nicotine dependence studies, utilizing its well-defined neuroconnectome as a robust platform, and to unravel the genetic basis of nicotine-motivated behaviors, we established the nicotine conditioned cue preference (CCP) paradigm. Nicotine CCP enables the assessment of nicotine preference and seeking, revealing a parallel to fundamental aspects of nicotine-dependent behaviors observed in mammals. We demonstrated that nicotine-elicited cue preference in worms is mediated by nicotinic acetylcholine receptors and requires dopamine for CCP development. Subsequently, we pinpointed nAChR subunits associated with nicotine preference and validated human GWAS candidates linked to nicotine dependence involved in nAChRs. Functional validation involves assessing the loss-of-function strain of the *CACNA2D3* ortholog and the knock-out (KO) strain of the *CACNA2D2* ortholog, closely related to *CACNA2D3* and sharing human smoking phenotypes. Our orthogonal approach substantiates the functional conservation of the α2δ subunit of the calcium channel in nicotine-motivated behavior. Nicotine CCP in *C. elegans* serves as a potent affirmation of the cross-species functional relevance of GWAS candidate genes involved in nicotine seeking associated with tobacco abuse, providing a streamlined yet comprehensive system for investigating intricate behavioral paradigms within a simplified and reliable framework.

## 1. Introduction

Tobacco abuse has been a major public health concern, and smoking remains a leading cause of preventable death [1]. The heritability of tobacco abuse is considerable, and nicotine dependence is a hallmark of its progress and maintenance [2,3,4,5,6]. Consequently, unraveling the genetic mechanisms underlying nicotine-motivated behavioral traits is an important strategy for understanding the underpinning mechanism of nicotine dependence. Human population genetics has identified statistically significant gene variants relevant to nicotine dependence [7,8,9]. Genome-wide association studies (GWASs) have successfully identified numerous Single-Nucleotide Polymorphisms (SNPs) associated with substance use disorder (SUD) over the past decade [9,10,11,12,13,14], but most candidate genetic variants have not been independently validated, nor have they improved our understanding of nicotine dependence.

We, therefore, exploit the rapid genetic workflow of *C. elegans*, which has a simple nervous system but completely defined connectome [15,16,17], as a tool for accelerating the functional validation of GWAS candidates associated with smoking/nicotine self-administration behavior. *C. elegans* responds to abused substances in a way that mimics substance-dependent behaviors observed in mammals [18,19,20,21,22,23,24,25,26,27]. Hence, worms have been a powerful model for SUD and nicotine dependence. *C. elegans* exhibits nicotine-responsive behaviors that resemble those observed in mammals, including nicotine withdrawal-dependent locomotion and the state-dependent development of chemical preference [22,26,27]. We implemented a nicotine conditioned cue preference (CCP) assay to measure the nicotine preference and seeking in *C. elegans*. The CCP assay stably elicits the acquisition, progress, and extinction of nicotine-paired cue preference and reveals properties mediated by nicotinic acetylcholine receptors (nAChRs) and dopamine, similar to mammals. Subsequently, we subjected nAChR mutant animals to the CCP assay to discern the specific nAChR subunits influencing nicotine seeking. Nicotine exerts its effects by binding to nAChRs, which are pentameric transmembrane proteins composed of α2, α4, α7, α10, and β2–β4 subunits [28]. Conformational transitions after binding to nicotine, accompanied by various regulatory mechanisms, enable nAChRs to respond dynamically to genetic and environmental factors. These receptors are critical for nicotine dependence as they stimulate synaptic activity in key brain regions, including the hippocampus, amygdala, ventral tegmental area (VTA), and nucleus accumbens (NAc). Of the nAChR subtypes found in the mammalian brain, the α7 homo-oligomer and α4β2 hetero-oligomer are the most abundant and have been implicated in regulating nicotine dependence. The further elucidation of the specific subunits involved in preference and seeking behavior elicited by nicotine will provide insight into the conservative role of nAChRs in mammals. In this context, previously reported nAChR subunits, identified as crucial for nicotine withdrawal-induced locomotion stimulation and associated with associative learning and rewarding effects in *C. elegans* [23,29], have been substantiated for their noteworthy involvement in nicotine CCP. Moreover, we demonstrated additional nAChR subunits contributing significantly to nicotine CCP. Subsequently, we delved into the GWAS candidates linked to human smoking and nicotine dependence, with a specific emphasis on the role of nAChR subunits. The nAChRs mediate conserved mechanisms governing nicotine-induced calcium dynamics [23,30,31,32], implicating the involvement of the Voltage-Gated Calcium Channel (VGCC). In our study, we performed the functional validation of α2δ subunits of the VGCC, previously highlighted in human GWAS studies for their association with nicotine dependence related to smoking. Our findings suggest that the α2δ subunit of the VGCC assumes a crucial role in nicotine preference, impacting the progression of nicotine dependence.

## 2. Results

### 2.1. Establishment of CCP (Conditioned Cue Preference)

**Figure 1:**
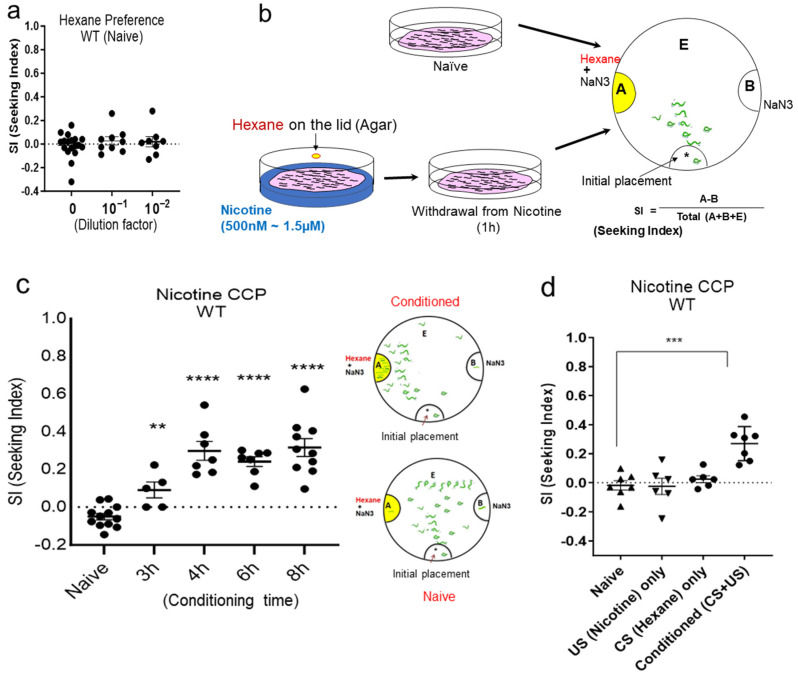
Nicotine conditioned cue preference (CCP) using hexane as Conditioned Stimulus (CS). (**a**) Identification of hexane as a neutral odor substance to naïve animals. One-way ANOVA of chemotaxis in wild-type animals to various concentrations of hexane did not show significant differences (*p* = 0.6136, F(2, 32) = 0.02649). (n ≥ 8) (**b**) The diagram of nicotine conditioned cue preference (CCP) using hexane as Conditioned Stimulus. Adult wild-type worms, aged one day, were pre-incubated with 1.5 µM nicotine and 2 µL of non-diluted hexane for conditioning. The conditioned worms were transferred to OP50-bacteria-seeded plate; then, 1 h later, worms withdrawn from nicotine were moved to the chemotaxis assay plate. A, representing the ethanol area; B, representing the control area; E, representing the neutral area. The Seeking Index (SI) is calculated as {[number of animals at A] − [number of animals at B]} divided by the total number of animals at areas A, B, and E. (**c**) Wild-type *C. elegans* develops CCP after chronic conditioning and following withdrawal from nicotine. One-way ANOVA, *p* < 0.001, F (4, 36) = 21.61, post hoc multiple comparison test; Dunnett’s (*p* < 0.01, **; *p* < 0.0001, ****). (n ≥ 7) (**d**) The CCP development by nicotine conditioning was validated by pretreatment of US only or CS only. US only, 4 h treatment of nicotine alone (1.5 µM); CS only, 4 h treatment of hexane alone; Conditioned (US + CS), 4 h conditioning of nicotine (1.5 µM) and hexane, all of which were withdrawn for 1 h before chemotaxis to hexane. Each dot represents trial of population assay. One-way ANOVA, *p* < 0.0001, F (3, 22) = 12.45, post hoc multiple comparison test; Dunnett’s (*p* < 0.001, ***). (n ≥ 7).

Adapting the mammalian Conditioned Place Preference (CPP) [33,34,35], a form of associative learning used to study the rewarding effects of drugs, we developed nicotine CCP and determined nicotine preference and seeking in *C. elegans*. Initially, we identified hexane, an alkane volatile odorant, as a suitable neutral stimulus for conditioning. Despite testing a spectrum of hexane concentrations, a discernible preference for hexane was not observed in *C. elegans* at the investigated levels (Figure 1a). Subsequently, we embarked on classical conditioning, utilizing nicotine as an Unconditioned Stimulus (US) (Figure 1b), grounded in insights garnered from preceding studies that explored behavioral and physiological responses across various nicotine concentrations [23]. Notably, *C. elegans* exhibits an initial response to nicotine, marked by heightened motility at an approximately 1.5 μM concentration [26], although concentrations as high as 100 µM induce locomotor paralysis, attributed to the acute activation of acetylcholine-sensitive ion channels in the worm’s motor neurons and muscles [23]. Moreover, *C. elegans* exhibits motivated behaviors induced by nicotine across a spectrum of concentrations, as detailed in previous studies [29]. Additionally, withdrawal from nicotine manifests as an enhanced stimulated locomotion, acting as a pronounced symptom in the context of nicotine withdrawal. Therefore, we applied various concentrations, including a 1.5 μM concentration, to induce nicotine-dependent locomotor stimulation and conducted our CCP assays accordingly. Wild-type animals successfully developed the acquisition of nicotine CCP in a time-dependent manner (Figure 1c) and successfully elicited preference even at concentrations higher than 1.5 μM but not paralytic. The Seeking Index (SI) was obtained, as represented in Figure 1b, with a high SI indicating that the nicotine-paired cue acted as a strong attractant, corresponding to the development of preference via conditioning with the reinforcing drug. Crucially, CCP did not manifest when the Unconditioned Stimulus (US) or Conditioned Stimulus (CS) was presented in isolation, affirming that conditioning occurred and facilitated CCP solely when the CS was paired with the US (Figure 1d).

**Figure 2:**
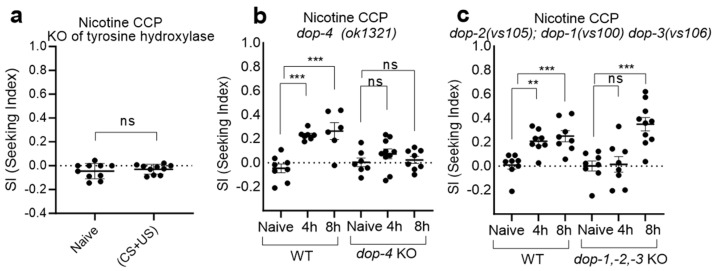
CCP is mediated via dopamine signaling. (**a**) Dopamine is required to develop CCP. A *cat-2* encodes a tyrosine hydroxylase, which catalyzes the conversion of tyrosine to L-DOPA, the biosynthetic precursor of dopamine. Conditioned (US + CS), 4 h conditioning of nicotine (1.5 µM) and hexane; animals were withdrawn for 1 h before chemotaxis to hexane. Each dot represents trial of population assay (n ≥ 10). ns, *p* = 0.7527 (Mann–Whitney test). (**b**) *dop-4(ok1321)* exhibited impaired CCP development compared to wild-type (ns, one-way ANOVA, F(2, 22) = 1.001, ns from post hoc multiple comparison test; Dunnett’s). Concurrently, wild-type CCP was rigorously evaluated at each trial, serving as a control for assessing the nicotine plate and conditioning process (*p* < 0.0001, one-way ANOVA, F(2, 18) = 16.48, *** represents *p* < 0.001 from post hoc multiple comparison test; Dunnett’s). Each dot represents trial of population assay (n ≥ 8). (**c**) The triple mutant strain *dop-2(vs105)*; *dop-1(vs100) dop-3(vs106)*, characterized by the combined deficiency of *dop-1*, *dop-2*, and *dop-3*, exhibited delayed CCP development in comparison to the wild-type (*p* = 0.0001, one-way ANOVA, F(2, 23) = 13.52, ns and *** from post hoc multiple comparison test; Dunnett’s). Concurrently, wild-type CCP was evaluated at each trial, serving as a control for assessing the nicotine plate and conditioning process (*p* = 0.0005, one-way ANOVA, F(2, 21) = 11.16, ** represents *p* < 0.01 and *** represents *p* < 0.001 from post hoc multiple comparison test; Dunnett’s). Each dot represents trial of population assay n ≥ 8).

Furthermore, we demonstrated that dopamine signaling mediates CCP induced by nicotine (Figure 2). The impairment of CCP development was prominently observed in KO mutant animals of *cat-2*, tyrosine hydroxylase in *C. elegans*, which was dopamine deficient (Figure 2a). In mammals, nicotine increases the firing rate of midbrain dopamine neurons by stimulating α4β2nAChRs, promoting nicotine dependence via the dopamine receptor [36]. Dopamine receptors are classified into two subfamilies: D1-like (D1 and D5) and D2-like (D2, D3, and D4). In mammals, nicotine-induced CPP is associated with the elevation of D1 and D2 receptor levels in both NAc and CA1 regions and mediated via both D1- and D2-like dopamine receptors [37]. Correspondingly, we extended our investigation to assess the dopamine receptors in *C. elegans*, revealing that *dop-4*, the D1-like dopamine receptor [38,39,40], exhibited the impaired development of CCP (Figure 2b). In concordance, although the deficiency of the remaining three dopamine receptors resulted in the delayed development of CCP, intact DOP-4 eventually induced successful CCP (Figure 2c). This highlights the integral role of *dop-4* in CCP manifestation, underscoring its significance within the dopaminergic signaling cascade.

**Figure 3:**
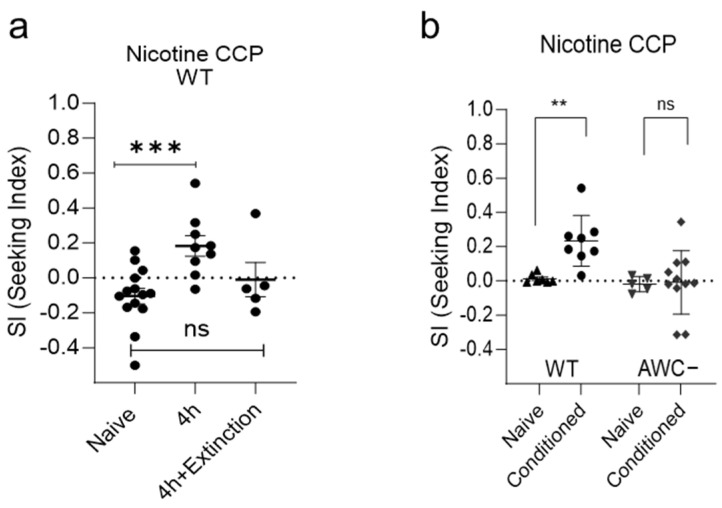
Characteristics of CCP. (**a**) Wild-type *C. elegans* learns extinction of CCP. Each dot represents trial of population assay. ns, *p* > 0.05; ***, *p* < 0.001 (Mann–Whitney test). (**b**) Nicotine conditioned cue preference (CCP) of AWC-neuron-ablated animals. Single session of 4 h CCP on 1.5 µM nicotine plates. Single session of 4 h CCP on 1.5 µM nicotine plates. ns, *p* > 0.05; **, *p* < 0.01; (Mann–Whitney test).

Wild-type animals can represent the extinction of CCP, greatly reducing paired rewarding. The expression of nicotine-induced CCP was abolished in subsequent chemotaxis assays after the presentation of CS (hexane) alone in the absence of US (nicotine) during the withdrawal period (Figure 3a), feasibly suggesting that CCP can be used to investigate the genes and pathways associated with reinstatement. To further investigate the mechanism involved in the regulation of CCP in the neural circuits, we explored the neural circuits mediating the acquisition of positive valence towards the Conditioned Stimulus (CS), which, post-conditioning, transitioned from being neutral to acting as an enticing cue. Chemotaxis behaviors are regulated primarily by the chemosensory neurons and modulated by the integration of signaling with interneurons [41,42]. *C. elegans* have 32 presumed chemosensory neurons that detect a variety of olfactory and gustatory cues [43,44,45,46]. In worms, AWC and AWA, ciliated chemosensory neurons, mediate attraction to the volatile odorants [47]. We exploited AWC-ablated animals to test in CCP. Killing a pair of AWC neurons via the expression of reconstituted Caspase [48,49] resulted in impaired CS (hexane) preference after conditioning with US (nicotine) (Figure 3b), suggesting that putative primary sensory neurons are AWC head neurons for attraction to hexane after conditioning.

**Figure 4:**
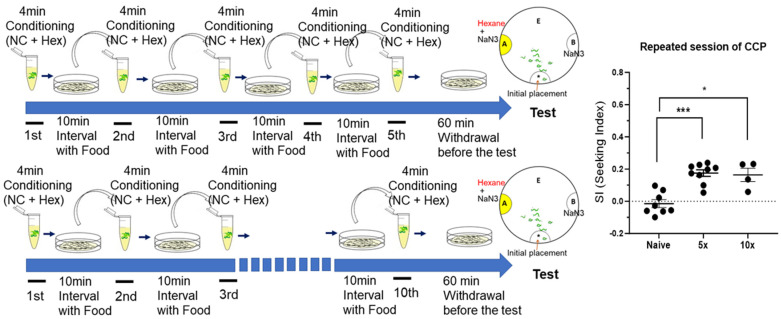
CCP is specifically elicited by nicotine. The short time of repeated conditioning (1 min, without food during conditioning) and withdrawal elicits successful CCP. A conditioning session (nicotine and hexane) was 1 min, and 10 min of withdrawal was followed. After multiple sessions of conditioning, the last withdrawal session was consistent as 60 min before conducting chemotaxis to CS. Each dot represents trial of population assay. *, *p* < 0.05; ***, *p* < 0.001 (Mann–Whitney test). (n ≥ 8).

In the laboratory, *C. elegans* was reared on agar plates enriched with OP50 bacteria as a primary nutritional source. To address potential concerns regarding the cultivation environment’s impact on the CCP assay, typically conducted under odor/starvation conditioning paradigms for Conditioned Place Aversion (CPA), we deliberately maintained the presence of OP50 bacteria during the nicotine conditioning and withdrawal processes in the CCP assay (Figure 1, Figure 2 and Figure 3). This strategic measure aimed to ensure the robustness and consistency of the experimental conditions. Nevertheless, to substantiate the specificity of the reinforcing effects of nicotine in *C. elegans* and to discern any potential influence of the food source, we extended the CCP paradigm to conditions devoid of OP50 bacteria. Notably, the development of CCP was still evident through repeated intermittent pairings of hexane with nicotine (Figure 4). Importantly, CCP was successfully induced by the short period of multiple conditioning sessions involving the Unconditioned Stimulus (US) (nicotine) and Conditioned Stimulus (CS) (hexane) in the absence of *E. coli* (food), reinforcing that CCP was specifically elicited by nicotine alone. This, coupled with the findings in Figure 1d, where CCP failed to establish when the CS (hexane) was presented alone with food, distinctly demonstrates that nicotine serves as the primary reinforcer in the progression of CCP in *C. elegans*.

### 2.2. CCP via nAChRs

**Figure 5:**
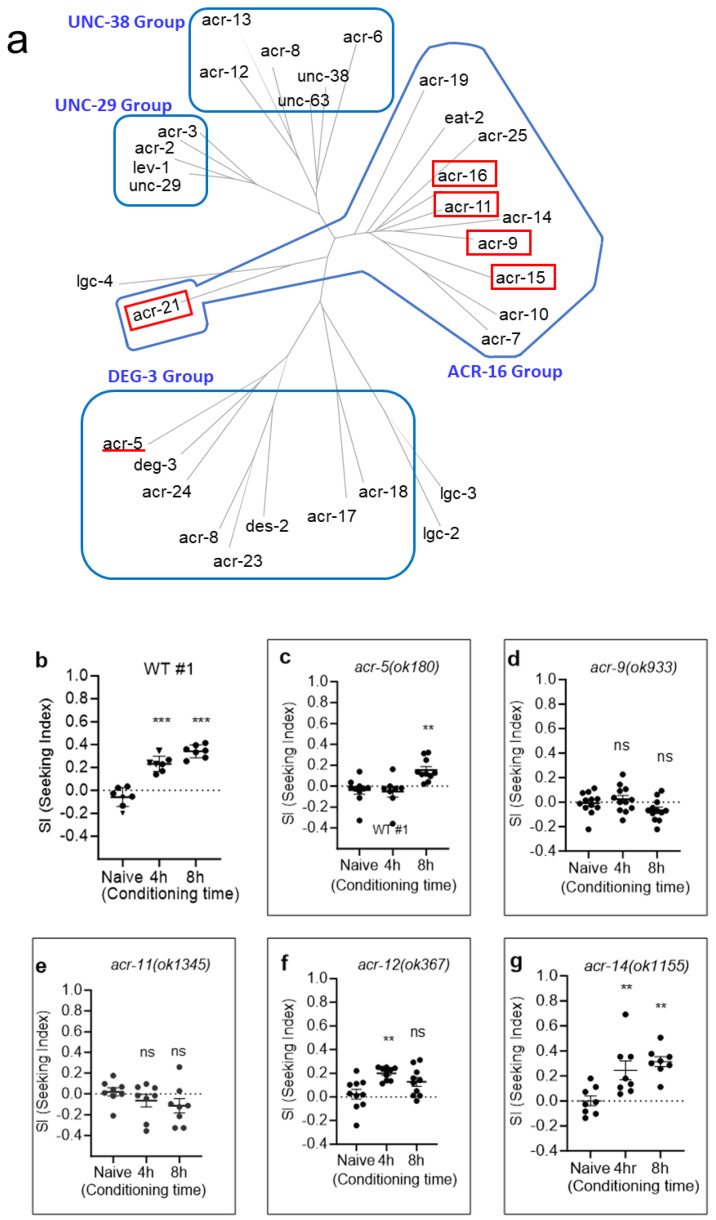

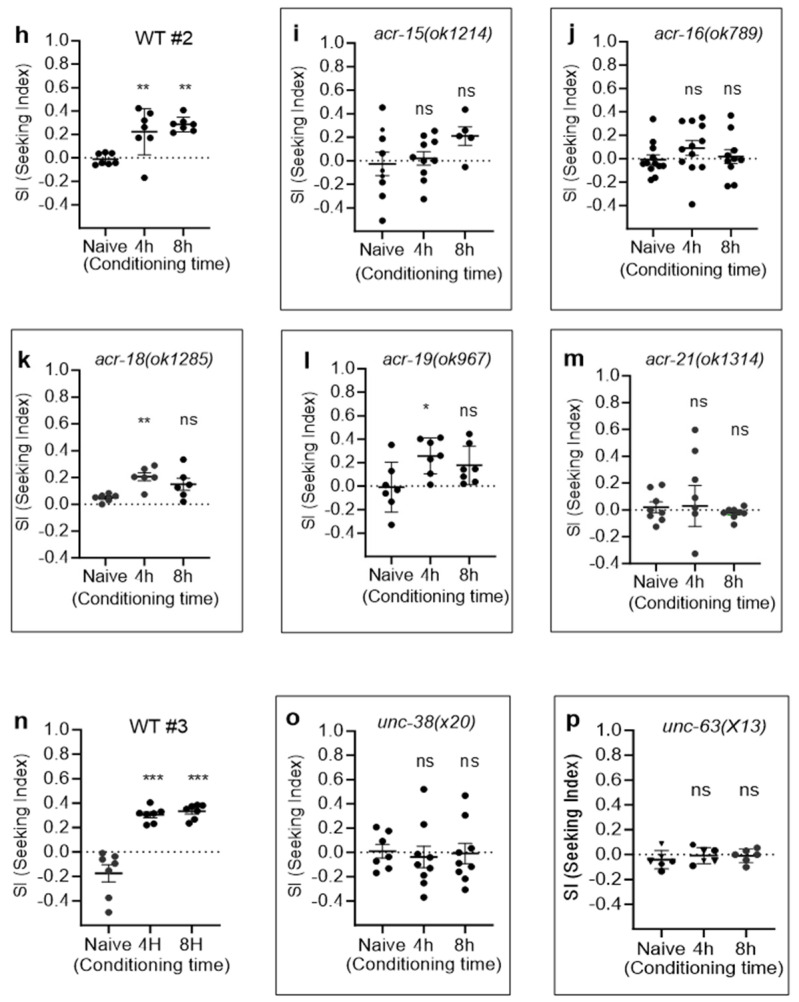
Identification of nAChRs relevant to CCP progression. (**a**) Phylogenetic analysis showing the nAChR receptor family of *C. elegans*. Using protein sequence homology, nAChR subunits were classified. (**b**) WT#1; *p* < 0.001, one-way ANOVA, F(2, 18) = 62.13, (*** represents *p* < 0.001 from post hoc multiple comparison test; Dunnett’s). (**c**) *acr-5 (ok180)*, *(p* = 0.002, one-way ANOVA, F(2, 23) = 8.396, ** represents *p* < 0.01 from post hoc multiple comparison test; Dunnett’s). (**d**) *acr-9 (ok933)*, (*p* = 0.08, one-way ANOVA, F(2, 33) = 2.755, ns, *p* > 0.05 from post hoc multiple comparison test; Dunnett’s). (**e**) *acr-11(ok1345)*, (*p* = 0.26, one-way ANOVA, F(2, 21) = 1.423, ns from post hoc multiple comparison test; Dunnett’s). (**f**) *acr-12(ok367)*, (*p* = 0.003, one-way ANOVA, F(2, 27) = 7.029, ns, *p* > 0.05; ** represents *p* < 0.01 from post hoc multiple comparison test; Dunnett’s). (**g**) *acr-14(ok1155)*, (*p* = 0.001, one-way ANOVA, F(2, 21) = 9.189, ** represents *p* < 0.01 from post hoc multiple comparison test; Dunnett’s). (**h**) WT #2, (*p* < 0.001, one-way ANOVA, F(2, 18) = 11.17, ** represents *p* < 0.01 from post hoc multiple comparison test; Dunnett’s). (**i**) *acr-15 (ok1214)*, *(p* = 0.20, one-way ANOVA, F(2, 21) = 1.735, ns from post hoc multiple comparison test; Dunnett’s). (**j**) *acr-16 (ok789)*, (*p* = 0.41, one-way ANOVA, F(2, 31) = 0.9130, ns from post hoc multiple comparison test; Dunnett’s). (**k**) *acr-18(ok1285)*, *(p* = 0.01, one-way ANOVA, F(2, 15) = 6.177, ns, *p* > 0.05; ** represents *p* < 0.01 from post hoc multiple comparison test; Dunnett’s). (**l**) *acr-19(ok967)*, (*p* = 0.03, one-way ANOVA, F(2, 18) = 4.080, ns, *p* > 0.05; * represents *p* < 0.05 from post hoc multiple comparison test; Dunnett’s). (**m**) *acr-21(ok1314)*, (*p* = 0.91, one-way ANOVA, F(2, 21) = 0.09735, ns from post hoc multiple comparison test; Dunnett’s). (**n**) WT#3, (*p* < 0.001, one-way ANOVA, F(2, 18) = 40.66, *** represents *p* < 0.001 from post hoc multiple comparison test; Dunnett’s). (**o**) *unc-38(x20)*, (*p* = 0.92, one-way ANOVA, F(2, 22) = 0.08521, ns, *p* > 0.05 from post hoc multiple comparison test; Dunnett’s). (**p**) *unc-63(x13)*, (*p* = 0.61, one-way ANOVA, F(2, 15) = 0.5035, ns, *p* > 0.05 from post hoc multiple comparison test; Dunnett’s).

Functional nAChRs are homopentameric or heteropentameric channels composed of five subunits by a combination of the α(α2–α10) and β(β2–β4) subunits [28,50,51,52]. We tested nAChR subunit KO mutant animals in CCP assay. A total of 29 nAChR homologs are reported in the *C. elegans* genome, whereas 17 are reported in mammals [23,53]. These nAChRs were classified into five groups, which were the ACR-16 group, UNC-29 group, UNC-38 group, ACR-8 group, and DEG-3 group [53]. We screened 12 nAChR mutants by the CCP assay, focusing on the ACR-16 group, which closely resembles the mammalian α7-nAChR subunit, a predominant subtype in the brain [54,55]. Here, we present consistent results with previous findings and also newly identified additional nAChR subunits associated with nicotine-induced motivated behaviors (Figure 5). In a single session of chronic CCP analysis, we identified the delayed development of CCP in KO mutants of *acr-5*, and it was impaired in *acr-15* and *acr-16*, which is compatible with previous reports on the nicotine-dependent locomotion of worms (Figure 5). Furthermore, we also identified the impaired development of CCP in KO mutants of *acr-9*, *acr-11*, and *acr-21* (Figure 5). The expression enrichment profile, provided by a single-cell gene expression profile of every neuron type in *C. elegans* (CeNGEN) [56], shows that *acr-9* is expressed in AVA, a crucial interneuron validated for the development of nicotine-dependent locomotion, in which *acr-15* and *acr-16* are expressed [23]. Recently, the AVA interneurons have been shown to participate in the integration of sensory-motor input and decision making [57]. Interestingly, *acr-21*, the nAChR α9 (CHRNA9) ortholog, is enriched in the RMG [56], the gap junctional hub interneurons that electrically connect to many sensory, motor, and interneurons, and is known to modulate pheromone attraction and social behavior [58]. RMG neurons form a close connection with AVA and ADA neurons, and *acr-11*, which we newly identified to play a role in nicotine CCP, is reported to be enriched in ADA. We also identified that the *unc-63* and *unc-38* mutants were defective in the development of CCP. This result is consistent with previous investigations into the nicotine-dependent stimulation of locomotion; however, a further comprehensive analysis will be required as both mutant animals, *unc-63* and *unc-38*, are not severely uncoordinated as described [23,59,60]. Nonetheless, our results demonstrate that the nicotine-elicited conditioned cue preference is mediated by nAChRs.

### 2.3. Orthogonal Test for Nicotine Preference

**Figure 6:**
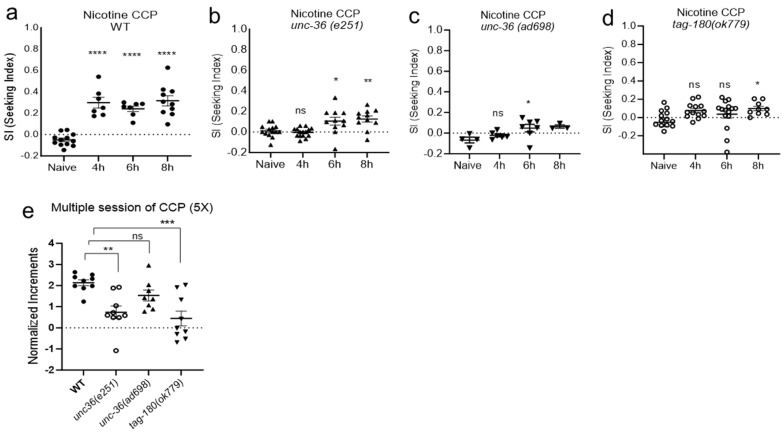
The orthogonal test evaluated nicotine preference of α2δ proteins. (**a**) Wild-type CCP was conducted in each trial to evaluate the drug plate and the conditioning process (*p* < 0.0001, one-way ANOVA, F(3, 32) = 27.17, **** represents *p* < 0.0001 from post hoc multiple comparison test; Dunnett’s). (N ≥ 7) (**b**) *unc-36 (e251)*, ortholog of *CACNA2D3*, showed delayed and reduced development of CCP (*p* = 0.0003, one-way ANOVA, F(3, 47) = 7.694, ns, *p* > 0.05; * represents *p* < 0.05 and ** *p* < 0.05 from post hoc multiple comparison test; Dunnett’s). (n ≥ 11) (**c**) Impaired CCP was observed in *unc-36 (ad698)*, ortholog of *CACNA2D3* (*p* = 0.0409, one-way ANOVA, F(3, 16) = 3.475, ns, *p* > 0.05; * represents *p* < 0.05 from post hoc multiple comparison test; Dunnett’s). (n ≥ 7) (**d**) Impaired CCP in *tag-180 (ok779)*, ortholog of *CACNA2D2*. (*p* = 0.0455, one-way ANOVA, F(3, 47) = 2.885, ns, *p* > 0.05; * represents *p* < 0.05 from post hoc multiple comparison test; Dunnett’s). (n ≥ 9) (**e**) Orthogonal test in repeated CCP. Repeated training of conditioning and intermittent withdrawal further demonstrated reduced development of nicotine preference in the mutant animals of α2δ orthologs. (*p* = 0.0004, one-way ANOVA, F(3, 31) = 8.112, ns, *p* > 0.05; ** represents *p* < 0.01 and *** *p* < 0.001 from post hoc multiple comparison test; Dunnett’s). (n ≥ 8) Each dot represents a trial of population assay.

The cross-species functional validation of GWAS candidates using *C. elegans* has been used successfully to demonstrate the functional relevance of candidates in substance-dependent behaviors [61]. We asked whether nicotine CCP in worms could be a viable and useful tool to accelerate the assessment of biologically significant pathways associated with nicotine dependence through the rapid functional characterization of GWAS candidates. Nicotine has been reported to evoke a calcium response from worms to mammals [23,30,31,32]. The nAChRs mediate increased intracellular calcium via VGCC-dependent and VGCC-independent manners that contribute to neural plasticity. The genome-wide meta-analysis on nicotine dependence has reported the protective role of *CACNA2D3* in nicotine dependence for African Americans [62]. *CACNA2D3* is also reported in the association of success in abstaining from smoking [63]. *CACNA2D3* is responsible for encoding the α2δ, auxiliary subunits of the VGCC, which influence the biophysical properties of the calcium channels [64]. The worm ortholog of *CACNA2D3* modulates voltage dependence, activation kinetics, and the conductance of calcium currents of the VGCC like mammalian a2δ [65]. Other members of the α2δ family, such as *CACNA2D2*, are also associated with nicotine dependence, smoking initiation, and cigarette consumption [66]. The loss-of-function alleles of *unc-36*, the *CACNA2D3* ortholog, were tested in CCP for the functional validation in the development of nicotine preference. We tested multiple mutant alleles of *unc-36*. *unc-36 (e251)* and *unc-36 (ad698)* are both loss-of-function alleles by the introduction of the premature stop codon and showed the delayed or impaired progress of nicotine-conditioned cue preference in a single session of chronic CCP, unlike WT animals (Figure 6a–c). We also tested mutant animals of *tag-180*, the *CACNA2D2* ortholog, which is closely related to *CACNA2D3* in the same family and shares the human smoking phenotypes. The *tag-180 (ok779)* deletion mutant (KO) showed the impaired development of nicotine preference (Figure 6d). We also tested animals in a repeated session of conditioning and intermittent withdrawals. The orthogonal test exhibited a reduced development of CCP in *unc*-*36 (e251)* and *tag-180(ok779)* (Figure 6e). Taken together, our data demonstrate that the α2δ subunit of the VGCC is required for a nicotine preference, contributing to the development of nicotine dependence.

## 3. Discussion

The progression of dependence represents a complex brain disorder that disrupts the functioning of key neural circuits. Nicotine induces various functional and structural neuroadaptive changes within the central nervous system, particularly within the reward circuits that play a crucial role in motivation and associative learning. The reward circuit has evolved to reinforce pivotal behaviors for survival, such as feeding and reproduction. Dopamine, a primary neurotransmitter driving this process, undergoes changes in dopaminergic transmission crucially associated with the development of dependence. In *C. elegans*, dopamine is involved in food-searching behavior, ensuring that worms remain in food-abundant areas, thereby increasing their chances of survival in nature [67,68]. In this context, dopamine in *C. elegans* has been known to play a central role in regulating the motor circuit, akin to the significant role of dopamine in modulating basal ganglia circuits for fine-tuning motor control in mammals [69,70].

Since the initial discovery of positive reinforcing circuits in the brain, where electrical stimulation triggered vigorous lever-pressing behavior [71], it has been clear that this phenomenon is closely linked to a surge in dopamine levels within the ventral tegmental area (VTA) and nucleus accumbens (NAc) [72,73]. This surge in dopamine plays a pivotal role in reinforcing substance conditioning in the dorsal striatum, directing attention toward cues predicting substance availability and intensifying the motivation to obtain the substance. Consequently, the drive to seek the substance precedes its consumption and is triggered by exposure to cues predicting its availability. In extending our investigation to *C. elegans*, we demonstrate that dopamine signaling plays a pivotal role in reinforcing nicotine conditioning in this model organism. Our findings represent a parallelism comparable to mammalian systems, emphasizing the evolutionary conservation of dopamine-mediated reinforcement mechanisms. Furthermore, this is compatible with our previous study, affirming that *C. elegans* recapitulates mammalian alcohol preference properties mediated by dopamine [22]. The convergence of these observations underscores the significance of *C. elegans* as a model organism for unraveling conserved neurobiological pathways implicated in substance-seeking behaviors, offering a reliable tool for the cross-species functional validation of targets linked to nicotine-seeking behavior and the exploration of pathways involved in the pathology of tobacco abuse.

The *C. elegans* genome contains four dopamine receptors, comprising two D1-like receptors, namely *dop-1* and *dop-4*, and two D2-like receptors, denoted as *dop-2* and *dop-3* [38,74]. The *cat-2* and *dop-4* KO mutants manifested the compromised development of conditioned cue preference (CCP), thereby underscoring the pivotal role of the dopaminergic signaling cascade, particularly exemplified by the significance attributed to the DOP-4 receptor. Our findings additionally unveiled a deferred progression of CCP in mutants lacking the other three dopamine receptors: *dop-1*, *dop-2*, and *dop-3*. Nevertheless, the unaltered functionality of DOP-4 ultimately resulted in the successful establishment of CCP, accentuating its central involvement in the reinforcement process induced by nicotine. The *dop-4* receptor has been implicated as a key player in amphetamine-induced Swimming-Induced Paralysis (SWIP), which is a behavioral response observed when *C. elegans* is placed in a liquid environment, particularly under stressful conditions like vigorous swimming [25]. Moreover, *dop-4* has been reported to contribute to the alcohol-induced disinhibition of behaviors, including alterations in crawling and escape responses. The discerned centrality of *dop-4* prompts further inquiries into the intricacies of specific neural circuits and potential downstream molecular pathways intricately associated with nicotine-dependent behaviors. While our investigation solidifies the prominence of DOP-4 in nicotine-induced CCP, the precise neural circuits and the subsequent molecular events await comprehensive elucidation. It is plausible that DOP-4 exerts its influence within the intricate framework of chemosensory and interneuronal circuits, actively contributing to the nuanced modulation of behavioral responses to nicotine.

Although human genetic association studies have been successful in revealing genetic factors and variants associated with smoking-related phenotypes, the estimated heritability has been limited to explaining underlying mechanisms. When analyzing the NHGRI/EBI GWAS catalog (release: 14 January 2021), it contained 1504 SNPs associated with smoking/nicotine that reached genome-wide significance. However, a substantial majority of these variants (93%) are not replicated by independent studies, lacking validation and novel insights into potential treatments. Experimental approaches for functional validation will be required to determine whether candidate genes have an actual role in the disease. Thus, various attempts have been suggested to effectively perform functional validation and comprehensive analysis [75]. The remarkable conservation of genes and behavioral traits associated with a higher susceptibility to mental disorders in *C. elegans* strengthens the applicability of this model organism in psychiatric research. In addition, the simplicity of the *C. elegans* system may contribute to implementing the fundamental circuit arrangement and associated pivotal modulation mechanisms that have been conserved throughout the evolution of more complex brains.

Cross-species functional validation, leveraging the distinctive attributes of worms, has consistently proven to be an invaluable tool in genetic study. For example, the introduction of a human TRPC (transient receptor potential canonical) channel can rescue the defective nicotine-dependent simulated locomotion phenotype of the worm TRPC channel KO strain [23]. A mammalian transient receptor potential channel vanilloid (TRPV) can substitute the worm ortholog and direct behavioral responses [76]. The transgenic worms containing the human SLC18A2 gene provided a model to investigate the brain dopamine and serotonin vesicular transport disease [77]. Recently, interspecies chimerism with a mammalian gene in the worm platform identified an orphan anti-opioid system [78]. The transgenic worm to express the mammalian μ (mu) opioid receptor (MOR), which is not normally found in the worm genome, responds to opioids such as morphine and fentanyl. Successively, this transgenic worm contributed to finding the orphan GPCR of which the mammalian ortholog shows functional conservation related to the anti-opioid pathway.

We exploited worms to define vulnerability phenotypes by the proper modeling of behavioral phenotypes and to test the functional evaluation of human GWAS candidates associated with nicotine dependence and smoking. We identified that nicotine-elicited cue preference is mediated by nicotinic acetylcholine receptors and dopamine signaling in worms. Collectively, our findings underscore that worms manifest pivotal features akin to nicotine-dependent behaviors observed in mammals. A previously identified GWAS variant of *CACNA2D3*, in which the SNP is in the intron region, was not prioritized for further validation, but it was reported that this variant was associated with reduced expression levels in three human brain tissues and was associated with nicotine dependence [62]. We validated its function by testing the loss-of-function or KO strains of the ortholog that allow for further pathway evaluation afterward. A *CACNA2D3* encodes α2δ, auxiliary subunits of the VGCC, that influence the biophysical properties of the calcium channels. VGCCs are pivotal in excitable cells with permeability to mainly calcium ions. Although it has been suggested that the permeation of calcium ions into cells through VGCCs will play a pivotal role in the induction of the plasticity of nicotine through nAChRs [30,79,80], close interaction between nAChRs and VGCCs for the subsequent event to mediate the nicotine response depends on the cell types, in which specific subtypes of nAChRs are expressed [51,52]. Mostly, non-α7-nAChRs mainly interact with the VGCC to mediate the signaling caused by nicotine.

α2δ proteins are encoded by four genes (*CACNA2D1*, *CACNA2D2*, *CACNA2D3*, *CACNA2D4*) and are expressed throughout the central nervous system to co-assemble with most of the α1 subunit, forming functional calcium channels [81]. α2δ proteins also interact with other proteins such as α-neurexins, LRP1 (low-density lipoprotein receptor-related protein 1), NMDA receptors (N-methyl- d-aspartate), and BK channels (large-conductance calcium-activated potassium channels) [82,83,84,85]. Some of these might be related to recent implications of α2δ proteins in the progress of SUD. Like *CACNA2D2* and *CACNA2D3* have been reported as GWAS candidates associated with nicotine dependence [62,66], CACNA2D1 has been involved in increased presynaptic NMDAR activity associated with hyperalgesia following chronic morphine [86]. Aberrant interaction between thrombospondin (TSP) and CACNA2D1 has been proposed as a possible mechanism of synaptic remodeling in the hippocampus during chronic ethanol consumption [87]. Interaction between α-neurexins and α2δ proteins is evolutionarily well conserved, endorsed by an interaction between NRX-1 and UNC-36 in *C. eleagns* [82]. The *C. elegans* genome includes two genes predicted to encode α2δ family proteins, *unc-36* and *tag-180*, predicted to be a *CACNA2D3/CACNA2D1*-like ortholog and *CACNA2D2*-like ortholog, respectively [88]. Like mammalian α2δ proteins, the function of UNC-36 in the modulation of the voltage dependence, the activation kinetics, and the conductance of calcium currents was electrophysiologically validated in the neuromuscular junction, whereas TAG-180 has no effects [65]. UNC-36 has been also demonstrated as a regulator of synaptogenesis together with UNC-2, a Ca_v_2-like α1 subunit of the VGCC, in the neuromuscular junction [89]. Interestingly, *tag-180* has not shown a functional association related to calcium channel activity so far. However, it is of interest that the behavioral phenotype of *tag-180* in nicotine-motivated behavior has been defined here. Perhaps this reflects the non-canonical interactions and role of α2δ proteins, such as the accumulation of CACNA2D2 in lipid rafts independently from the interaction with calcium channels [90].

This study introduces a novel CPP paradigm assay for nicotine seeking in worms, offering a robust tool for the functional validation of genes associated with nicotine dependence. Beyond its genetic applications, this tool holds potential for investigating the intricate interplay between genetic determinants and environmental influences in the context of nicotine dependence. The nicotine-seeking behavior determined through CPP, coupled with functional validation, revealed the involvement of orthologs of *CACNA2D2* and *CACNA2D3* in nicotine-motivated behavior in *C. elegans*. Subsequent studies on the α2δ protein should focus on a comprehensive functional characterization of the mechanisms underlying nicotine seeking and taking. Ongoing efforts aim to identify specific nAChR subunits influencing nicotine seeking and define the subset of neurons in which these subunits operate.

## 4. Materials and Methods

All strains were cultivated on nematode growth media (NGM) plates with the *Escherichia coli* strain OP50 at 20 °C as described [91], and the hermaphrodite worm was used for behavioral analysis. The Bristol N2 strain was used as wild-type (WT) animals. The strains below were obtained from Caenorhabditis Genetics Center (CGC, Minneapolis, MN, USA), which is supported by the National Institutes of Health Office of Research Infrastructure Programs (P40 OD010440).

The following mutant alleles were used in the study: *cat-2(e1112)*, *dop-4(ok1321)*, *dop-2(vs105)*, *dop-1(vs100)*, *dop-3(vs106)*, *acr-5(ok180)*, *acr-9(ok933)*, *acr-11(ok1345)*, *acr-12(ok367)*, *acr-14(ok1155)*, *acr-15(ok1214)*, *acr-16(ok789)*, *acr-18(ok1285)*, *acr-19(ok967)*, *acr-21(ok1314)*, *unc-38 (x20)*, *unc-63(x13)*, *unc-36(e251)*, *unc-36(ad698)*, and *tag-180(ok779)*. The strain PY7502, oyIs85[*ceh-36*p::TU#813 + *ceh-36*::TU#814 + *srtx-1*p::GFP + *unc122*p::DsRed], was used for AWC-ablated animals. PY7502 was generated via the expression of recCaspases (split caspases) [48] under *ceh-36* promoter [92].

### 4.1. Behavioral Assay

#### 4.1.1. Nicotine Conditioning

The nicotine plates were prepared freshly on a 60 mm plate before the conditioning assay. S-(-)-Nicotine (Thermo Scientific Chemicals, Waltham, MA USA) was diluted to concentrations of 100 μM and 1000 μM using sterilized double-distilled water. When NGM was cooled to 55 °C after sterilization, 100 μM of nicotine stock was added up to the designated concentration (1.5 μM) and poured into plates. Then, 100 μL of concentrated OP50 was seeded on the nicotine plates, and then, one day later, nicotine plates were used for conditioning. OP50-seeded nicotine plates were stored at 4 °C and consumed within a week for the conditioning.

The synchronized eggs were collected for 3 h and then were harvested with S basal-buffer (100 mM sodium chloride, 50 mM potassium phosphate (pH 6.0)) for the conditioning when they reached the Day 1, young adult stage (16–24 h later after the mid-L4 stage). To introduce hexane as a Conditioned Stimulus (CS) to the nicotine conditioning plate, 80 µL of agar lump (2% BBL agar) on the lid (60 mm plate) was freshly prepared before the conditioning. S basal-buffer harvested animals were placed in the middle of the conditioning plate (1.5 μM nicotine) and then covered with a lid with an agar lump, to which 3 µL of hexane (98.5% hexane as non-diluted) was added. Since the CS was a volatile odor, the plates were sealed with parafilm during conditioning. After 4, 6, or 8 h of conditioning (for 8 h, 3 µL of hexane was refilled to the agar lump after 4 h for another 4 h), worms were washed with S basal-buffer 3 times and then transferred to OP50-seeded NGM without nicotine and hexane for the withdrawal session. Next, 1 h later, a chemotaxis assay was conducted. The withdrawal procedure (here, 1 h) was followed after all the sessions, including [CS only] and [US only] which validated CCP, prior to performing chemotaxis to CS.

For the repeated conditioning session, we delicately executed conditioning by gently rotating *C. elegans* in 1 mL of S basal-buffer containing 1.5 μM of nicotine and 2 μL of hexane for 1 min. Subsequently, a total of 4 min was allocated, including 3 min during which worms were allowed to sink before washing commenced. After washing with S basal 3 times, conditioned worms were placed on OP50-seeded NGM for a 10 min withdrawal session. This short-duration conditioning cycle was reiterated at 10 min intervals, culminating in a final withdrawal period exceeding 1 h. Following this regimen, the chemotaxis assay to CS was performed to assess the acquired behavioral responses.

#### 4.1.2. Chemotaxis to CS

A chemotaxis assay was performed as described previously [43,46,93]. Briefly, 10 mL of chemotaxis media (1.6% BBL-agar, 5 mM potassium phosphate; pH 6.0, 1 mM CaCl_2_, 1 mM MgSO_4_) was prepared on a 100 mm Petri dish. Then, 1 µL of 100 mM NaN_3_ was added to the point marked in the section of A and B (Figure 1b). Next, 1 µL of CS (undiluted hexane) was added on top of the NaN_3_ in the section of A. Immediately after the CS was absorbed into the 100 mm chemotaxis plate, about 100 washed animals were placed in the area marked using a glassware micropipette. Then, 40 min later with parafilm sealing, the number of accumulated animals in each section marked (Figure 1b) was counted to calculate the Seeking Index. The index was calculated by [(number of animals in A—number of animals in B)/Total number of animals [Seeking index SI = (A − B)/Total(A + B + E)]. A total of 100–150 animals were tested in each trial to obtain the index.

In the case of uncoordinated strains (*unc-38 (x20)*, *unc-63(x13)*, *unc-36(e251)*, *unc-36(ad698)*), their CCP was confirmed again by creating an environment that could be reached to the CS (same concentration given) by moving a short distance. A square 100 mm chemotaxis plate with a grid engraved on it was prepared using the same amount of chemotaxis media. And then, chemotaxis was performed in a space where animals showing uncoordinated movement using only 60 mm in the center could arrive at their destination in time. At these trials, the WT control was also performed under the same conditions.

### 4.2. Statistical Analysis

WT control groups were always tested together in each trial to evaluate the drug plate and the conditioning process. Each dot in the graph represents the population assay in which about 100–150 animals were tested. The mean and standard error of the mean (SEM) were determined for all experimental parameters. The data were analyzed by employing the Mann–Whitney or Dunnett’s tests using GraphPad Prism software (version 8.0.1). Data points with *p*-values below 0.05 (*p* < 0.05) were considered to be significant.

### 4.3. Sequence Alignment

Protein sequences were analyzed by a database similarity search [94], and the multiple protein sequences were simultaneously aligned using COBALT, a constraint based alignment tool [95]. The phylogenetic tree was constructed by COBALT using the minimum evolution method. The sequences used for the phylogenetic tree analysis were as follows: P48182.1, Q93149.1, P54246.5, NP_491354.2, NP_495647.1, NP_001361818.1, NP_510285.2, NP_508692.3, NP_491906.1, AAG35183.1, NP_495716.1, NP_505206.2, NP_505207.1, NP_001023961.1, NP_506868.2, NP_001129756.1, NP_001367183.1, NP_001355515.1, NP_504024.2, NP_001379138.1, NP_001380111.1, NP_496959.1, NP_001255705.1, NP_509932.2, NP_492399.1, NP_491472.1, NP_491533.2, G5ECT0.1, NP_001255865.1, Q19351.5, NP_509556.4, NP_001023570.2.

## Data Availability

Data is contained within the article.
